# Social changes through the lens of language: A big data study of Chinese modal verbs

**DOI:** 10.1371/journal.pone.0260210

**Published:** 2022-01-04

**Authors:** Shan Wang, Ruhan Liu, Chu-Ren Huang

**Affiliations:** 1 Department of Chinese Language and Literature, Faculty of Arts and Humanities, University of Macau, Zhuhai, China; 2 Institute of Collaborative Innovation, University of Macau, Zhuhai, China; 3 Department of Chinese and Bilingual Studies, The Hong Kong Polytechnic University, Hong Kong, China; 4 The Hong Kong Polytechnic University-Peking University Research Centre on Chinese Linguistics, Hong Kong, China; The University of Hong Kong, HONG KONG

## Abstract

Leech’s corpus-based comparison of English modal verbs from 1961 to 1992 showed the steep decline of all modal verbs together, which he ascribed to continuing changes towards a more equal and less authority-driven society. This study inspired many diachronic and synchronic studies, mostly on English modal verbs and largely assuming the correlation between the use of modal verbs and power relations. Yet, there are continuing debates on sampling design and the choices of corpora. In addition, this hypothesis has not been attested in any other language with comparable corpus size or examined with longitudinal studies. This study tracks the use of Chinese modal verbs from 1901 to 2009, covering the historical events of the New Culture Movement, the establishment of the PRC, the implementation of simplified characters and the completion and finalization of simplification of the Chinese writing system. We found that the usage of modal verbs did rise and fall during the last century, and for more complex reasons. We also demonstrated that our longitudinal end-to-end approach produces convincing analysis on English modal verbs that reconciles conflicting results in the literature adopting Leech’s point-to-point approach.

## Introduction

Leech’s [1] corpus-based comparison of English modal verbs from 1961 to 1992 showed drops in the usage of all modal verbs that he ascribed to continuing changes towards a more equal and less authority-driven society. This study inspired many later diachronic and synchronic studies, mostly on English modal verbs and largely assuming the correlation between the use of modal verbs and power relations. Yet, there are continuing debates on the point-to-point sampling design on the choices of corpora. Most crucially, this hypothesis has not been extended to any other language with comparable corpus size or examined with longitudinal studies. Chinese not only allows for a chance to verify the hypothesis in a different language (especially one with a very conservative writing system) but also in a critical period with both multiple high impact historical events and a couple of significant language reform movements (the vernacular movement and the simplification of Chinese writing systems). It allows us a unique opportunity to observe the correlation between the usage of modality with both social, historical events and meta-linguistic events.

The availability of large-scale diachronic digitized corpora facilitates the corpus-based approach to the study of language changes [2–6]. Corpus-based approaches are not only typically based on strong empirical evidence, but also often rely on quantitative modeling [7]. The availability of historical corpora also allows end-to-end longitudinal studies to focus on a particular period of interest. In this context, historical corpora can be considered as perhaps the only continuous chronological databases of collective human behavior changes [8]. As such, quantitative research of language changes can be a useful tool to explore the impacts of historical events and social evolution [9]. Among linguistic cues, modal verbs are the main devices for expressing stances and power relations, as well as performing speech acts, which have been the foci of historical studies both from linguistic and social change perspectives [1, 10–13]. However, such studies have focused on English and a few European languages, typically covering less than 100 years and focusing only on a few words, and rarely adopt end-to-end longitudinal approaches [14–17].

Even though the study of English modal verbs has proven the correlation between social changes and language use, no such exploration has been conducted with Chinese. Thus, this paper aims to analyze the usage trends of Chinese modal verbs over a critical period in the development of the Chinese language and society. The major events that occurred in this period include the New Culture Movement during 1915–1923 that established the standard of writing in vernacular Chinese (instead of classical Chinese); the founding of the People’s Republic of China in 1949, the formal adoption of the simplified characters in 1956, and the completion and finalization of simplification of the Chinese writing system in 1986. Our data sampling takes each decade as a unit, ending with the year 2009. This sampling technique allows us to precisely observe the effect of the first three events (in 1919, 1949, and 1960), although not with such precision for the last two events. We believe that a separate study to focus on these more recent events with a shorter sample span (perhaps 10 years or shorter) is needed, given the faster pace of societal changes and the size of available data. Yet, interpretations of such studies will benefit greatly from the verification of methodology and the global view of the context provided by the current study.

This study of Chinese modal verbs in the last century has several important implications. It will be one of the first such studies based on a large diachronic non-English corpus and certainly the first on a Sino-Tibetan language. In addition, the longitudinal coverage of over 100 years provides a good test to show if the direction of usage changes remains constant or varies over time. Furthermore, since the Chinese language went through two major reforms in the past last century, this diachronic study gives us a unique opportunity to look at the potential influence of language reforms on language uses.

Finally, our new diachronic data-driven analysis aims to resolve the long-standing controversy due to conflicting results based reported in Leech [1] and Millar [11]. Leech [1] reported a general tendency of declining modal verbs. Yet, Millar [11] reported both increasing and decreasing trends that vary according to different modal verbs. Since both are corpus-driven descriptive studies, neither can be proven wrong on an empirical basis. Although Millar’s [11] criticism and Leech’s [9] rebuttal did suggest that the discrepancies likely arose from the differences in corpus sizes as well as whether the corpus is balanced or not. Still, understanding the source of discrepancy does not bring us closer to resolving the differences. This is another example of the well-known Galton’s problem [18], which states that empirically observable generalizations by themselves are not sufficient proof of an explanatory account. The fact that we have two conflicting sets of verifiable empirical generalizations makes it even more challenging to form a synthesizing account that could accommodate these two sets of generalizations.

We believe that the big data-driven longitudinal sampling approach may provide a solution to this challenge. On the one hand, to account for possible discrepancies in two different corpora, a study should rely on a dataset that has comprehensive coverage of both corpora. On the other hand, to reconcile conflicting previous observations of historical trends, a study must cover a longer period and with a finer granularity of data sampling. In other words, based on the premise that both generalizations are correct vis-à-vis each corpus, it is logical to assume that they either reflect the local trends specific to the particular subset of data or that the trends are an oversimplification due to a point-to-point comparison with a small number of time points. Thus, we propose to adopt the most comprehensive diachronic English corpus, namely, the Google Books corpus. Given the size and comprehensive sampling of the Google Books corpus over the same time period as the two corpora used in the previous studies, it is reasonable to assume that Google Books covers both. On the other hand, we propose to do an end-to-end longitudinal analysis of Google Books and for an extended period beyond both previous studies. This way we can ensure that the generalizations reported earlier can be subsumed by our more comprehensive study with year-to-year change patterns. In terms of solving Galton’s problem, we echo the approach proposed [19] for complex interactive systems. That is, we try to provide all corroborating evidence for the proposed account without attempting to establish a single logical causal relation.

In order to find out the differences in the use of modal verbs between English and Chinese and further reflect the historical changes of Chinese society through the lens of language, this study tackles the following research questions in this study by examining the usage patterns of modal verbs during the critical 109 years: (1) Do modal verbs in different languages share similar patterns of decline? (2) Do high, median, and low value modal verbs show different usage trends and why? (3) Can an end-to-end longitudinal study of language big data focusing on modal verbs reveal significant events and trends in history? and (4) Do modal verbs show different degrees of sensitivities to different historical events in their usage trends?

## Literature review

The interaction of social and linguistic changes is a central issue in both social and historical linguistics, especially in terms of how social evolutions are reflected in language changes [20]. Past studies focused on the modeling of linguistic changes [21], although many focused on either long [22, 23] or short-term lexical changes [8, 24–27]. An important emerging trend is to leverage stance to an attitudinal expression in language to monitor social changes [28]. Among the linguistic devices studied, modality and modal verbs are highly relevant, not only because of their role in expressing a speaker’s attitudes, but also because they are among the most frequent words and hence can be reliably found in all corpora.

Modality marks the speaker’s opinion or attitude towards the event described in the sentence [29–31]. Halliday [29] assigned three levels of values to English modal verbs according to their pragmatic functions: high value, median value, and low value. These values differ crucially according to the social context they are used in [31]. One possible view is that the value of a modal verb is defined according to how “coercing” it is from the speaker to the listener. Thus, the value of the modal verbs used can be treated as a cue to the power relation between the interlocutors. This function of modal verbs in a social context is a theoretical foundation for studying the correlation between changes of modal verb usages and social changes.

In Chinese grammatical studies, modal verbs are sometimes called modal auxiliary verbs [32–36] or simply modals or modal adverbs [37, 38], which largely overlap with what is traditionally called the *can-wish* (能愿 *néngyuàn*) verbs in literature [33]. Ma [32] further classified the Chinese *can-wish* verbs into six categories, with a special focus on the ordering and collocational behavior when two or more modal verbs occur together: Possibility Verbs A, Necessity Verbs, Possibility Verbs B, Wish (Inclination) Verbs, Evaluation Verbs, Permission Verbs. Although Ma’s [32] six classes classification of *can-wish* verbs are commonly adopted in linguistic literature in China, he never gave explicit criteria for the classification. Closer examination of Ma [32] as well as relevant data, shows that the last two categories, evaluation verbs and permission verbs, do not behave as modality verbs but as adverbials when they appear before other verbs. For example, the evaluation verbs (e.g. 值得 *zhíde* ‘be worthy of’) and permission verbs (e.g. 准许 *zhǔnxǔ* ‘permit’) have the meaning and behavior of typical clause-taking verbs. Thus, we will exclude them in our analysis. In addition, the high-median-low values are widely adopted and shown to be useful in the analysis of power relations in Chinese [39], although there are some variations in details.

Following Leech’s [1] pioneering work based on Brown family corpora, Millar’s [11] study based on a much larger but unbalanced TIME corpus raised several theoretical and methodological issues. In addition, the usage trends they found differ significantly, as shown in Table 1.

**Table 1:** Comparison of results from Leech [1] and Millar [11] (sorted according to the gap).

Modal Verbs	Leech change in diff % (1961~1991–2) of British English	Leech change in diff % (1961~1991–2) of American English	Leech average	Millar change in diff % (1920s–2000s)	gap
Can	2.20%	-1.50%	0.35%	113.40%	113.05%
could	2.40%	-6.80%	-2.20%	103.00%	105.20%
May	-17.40%	-32.40%	-24.90%	59.70%	84.60%
might	-15.10%	-4.50%	-9.80%	15.70%	25.50%
should	-11.80%	-13.50%	-12.65%	2.70%	15.35%
Will	-2.70%	-11.10%	-6.90%	7.70%	14.60%
would	-11.00%	-6.10%	-8.55%	0.10%	8.65%
ought (to)	-44.20%	-30.00%	-37.10%	-44.90%	-7.80%
Must	-29.00%	-34.40%	-31.70%	-48.40%	-16.70%
Shall	-43.70%	-43.80%	-43.75%	-95.60%	-51.85%
need (n’t)	-40.20%	-12.50%	-26.35%	N/A	N/A
Total	-9.50%	-12.20%	-10.85%	22.90%	33.75%

Note that Millar [11] observed an overall rising trend, contrary to the prediction and observation of Leech [1]. In addition, the majority of modal verbs are observed to have rising frequencies vs Leech’s [1] falling frequencies of almost all modal verbs. Among all modal verbs, *can*, *could*, and *may* group show the most drastic difference of contradicting directions and over 80% discrepancies. We will resolve this puzzle in our first study, which will also confirm the methodology for our main study on Mandarin modal verbs.

The general consensus on theoretical issues of the analysis of modal verbs and the correlation of their usages to social changes was formed after Leech’s [10] response and described earlier in this paper. The corpus choice and data sampling method issues, however, have important implications for our current study. In terms of corpus size and coverage, Millar [11] pointed out the advantage of scaling up, which has been empirically verified by recent corpus-driven studies, many adopting the Web-as-Corpus approach [40]. In addition, in studies of variations and changes, the density of sampling vs population is a critical issue as one can never be sure if the change between two sampling points is simple and monotonic, or complex and multi-dimensional. This can be viewed as another constant source of Galton’s problem; that is, we can never be sure if the change between two points of direct comparison is the result of a direct causal change or the sum of a series of changes. One possible solution to this issue is to have longitudinal continuous sampling for an end-to-end study. That is, one could design the study to examine all data based on fixed diachronic periods, thus ensuring that all data and all times are accounted for. The Google Books corpus is arguably the single most comprehensive corpus in the world and also supports longitudinal continuous sampling. The early version of the Google Books corpus contains 5,195,769 digitized books and over 500 billion words containing 4% of all books ever published. There are 13 billion Chinese words [41]. The 2012 version contains data from 8,116,746 books covering 6% of all books ever published [42]. Despite some limitations, such as the quality of metadata and occasional OCR errors, the Google Books corpus still offers new research potentials [43]. The comprehensive coverage of data and the continuous span in time makes the Google Books corpus well-suited to support the research approach described as “Big Data Aided Armchair Linguistics” [8], adopting the original description of corpus linguistics by Fillmore [44]. This corpus also helps to solve the challenge of lacking large enough historical corpora for diachronic studies of low-frequency words [45]. The Google Books corpus based research typically utilizes the Google Books Ngram Viewer to extract and observe diachronic tendencies, such as the analysis of the complexity of British and Chinese culture from the 1900s to 2000 [46], the examination of the increasing use of the seven taboo words in American culture coinciding with rising cultural individualism from 1950 to 2008 [47], and the changing usage trends and switching relations (from competition to co-development) of a pair of near synonyms, gaming and gambling, to corroborate socio-economical changes [8].

## Methodology

Three methodology related issues are discussed in this section, each supported with either preliminary analysis and/or a calibration study. The first involves the identification, classification, and assignment of values to Chinese modal verbs in a corpus. The second deals with the optimization of corpus selection and data preparation for diachronic studies of modal verbs, including a calibration study using the Google Books corpus focusing on reconciling the differences between Leech [1] and Millar [11]. Lastly, we describe the methodology adopted for the selection and processing of corpus data for the current study, based on the Google Books corpus from 1901 to 2009. We take a longitudinal continuous sampling approach and record the total usage of each modal verb for every 10 years (per decade). Thus, the data extraction method is similar to Millar [11], which Leech accepted in his response [10]. The longitudinal and continuous study design is crucial as it allows comprehensive tracking of changes throughout the study periods and avoids over-generalization based on simple point-to-point direct comparison.

## Modal verbs and their modality values in Chinese

Modality expresses the speaker’s judgment on the propositional content of a sentence. The values of a modal verb may vary according to the contextual meaning of the social events it is used for, which can range from an inclination to obligation [29].

It is non-controversial to assume that socio-cultural changes would affect the nature of interpersonal interaction. Given modal verbs’ crucial linguistic functions of marking modality, we hypothesize that the changes in interpersonal interactions will be reflected in language uses by the values of modal verbs. To explore this hypothesis, we adopt definition from Halliday [29] to classify modal verbs into three categories according to their values: (1) high value: modal verbs involving the obligation, responsibility, and necessity of the target event; (2) median value: modal verbs used to express the speaker’s will to the target event and the prediction of the occurrence of related events; (3) low value: modal verbs used to express the possibility of the occurrence or the ability to be completed of the target event. These power values indicate the strength of inter-personal interaction, from highly obligatory to weakly permissive. Halliday’s [29] classification of English modal verbs is shown in Table 2.

**Table 2:** The values of English modal verbs.

Value	Low	Median	High
Positive	can, may, could, might, dare	will, would, should, is/ was to	must, ought to, need, has/ had to
Negative	needn’t, doesn’t/ didn’t + need to, have to	won’t, wouldn’t, shouldn’t, isn’t/ wasn’t to	mustn’t, oughtn’t to, can’t, couldn’t, mayn’t, mightn’t, hasn’t/ hadn’t to

As there is no consensus on the list and scope of Mandarin modal verbs in the literature, we follow the standard pedagogical account in *Chinese Proficiency Vocabulary and Chinese Character Level Syllabus (Revised Edition)* [48], from which we selected 21 typical modal verbs: 必须 *bìxū* ‘must,’ 当 *dāng* ‘should,’ 得 *děi ‘have to*,*’* 该 *gāi* ‘ought to,’ 敢 *gǎn* ‘dare,’ 会 *huì* ‘be likely to,’ 肯 *kěn* ‘be ready to,’ 可能 *kěnéng* ‘be possible,’ 可以 *kěyǐ* ‘may,’ 乐意 *lèyì* ‘be pleased to,’ 能 *néng* ‘can,’ 能够 *nénggòu* ‘be able to,’ 想 *xiǎng* ‘want to,’ 须 *xū* ‘must,’ 要 *yào* ‘would,’ 应 *yīng* ‘should,’ 应当 *yīngdāng* ‘should,’ 应该 *yīnggāi* ‘ought to,’ 愿 *yuàn* ‘be willing,’ 愿意 *yuànyì* ‘be willing,’ 总得 *zǒngděi* ‘be bound to.’

To ensure a meaningful comparison with English modal verbs, these verbs are classified with the same three value categories. Our classification of Chinese modal verbs follows Ma [32] and covers the first four of his six-category system, after eliminating the two non-modality categories, as discussed earlier. In addition, Ma’s Possibility Verbs A contain a single verb 可能 *kěnéng* ‘be possible,’ and it is clear from Ma [32] that the main motivation for having two different possibility categories A and B is due to the special syntactic behavior of 可能 *kěnéng* ‘be possible.’ Hence we merge the two Possibility categories and deal with three sub-classes of Mandarin modal verbs in this study: Necessity, Possibility, and Inclination (wish), as shown in Table 3.

**Table 3:** Types of Chinese modal verbs (updated based on Ma [32]).

Categories	Modal Verbs
Necessity	得 *děi ‘have to’*, 应 *yīng* ‘should’, 该 *gāi* ‘ought to’, 应该 *yīnggāi* ‘ought to’, 应当 *yīngdāng* ‘should’, 须得 x*ūděi* ‘have to’, 必得 *bìděi ‘have to’*, 要*yào* ‘must’, 犯得着 *fàndézháo* ‘worthwhile’, 犯不着 *fànbuzháo* ‘no need to do’
Possibility	可能 *kěnéng* ‘be possible’, 会 *huì* ‘be likely to’, 可以 *kěyǐ* ‘may’, 能 *néng* ‘can’, 能够 *nénggòu* ‘be able to’, 好 *hǎo* ‘can’, 免不了 *miǎnbuliǎo* ‘inevitable’, 得以 *déyǐ* ‘be able to’, 容易 *róngyì* ‘might’, 来得及 *láidéjí* ‘still can’
Inclination	乐意 *lèyì* ‘be pleased to’, 愿*yuàn* ‘be willing’, 愿意 *yuànyì* ‘be willing’, 情愿 *qíngyuàn* ‘be willing’, 想 *xiǎng* ‘want to’, 要 *yào* ‘would’, 要想 *yàoxiǎng* ‘(if you) want to’, 希望 *xīwàng* ‘hope that’, 企图 *qìtú* ‘attempt to’, 好意思 *hǎoyìsi* ‘be willing’, 乐得 *lèdé* ‘be willing’, 高兴 *gāoxìng* ‘be pleased to’, 乐得 *lèdé* ‘be pleased to’, 高兴 *gāoxìng* ‘be pleased to’, 乐于 *lèyú* ‘be pleased to’, 肯 *kěn* ‘be willing’, 敢 *gǎn* ‘dare to’, 敢于 *gǎnyú* ‘dare to’, 勇于 *yǒngyú* ‘dare to’, 甘于 *gānyú* ‘be willing’, 苦于 *kǔyú* ‘suffer from’, 懒得 *lǎndé* ‘lazy to’, 忍心 *rěnxīn* ‘be willing’

Note that the Halliday-Hasan theory of power values of modal verbs predicts that necessity, possibility, and inclination (wish) would map to high, low and median values by default. However, as Ma’s classification relies more heavily on syntactic positions, some adjustments are needed. First, 敢 *gǎn* ‘dare’ is excluded from this study because it is not possible to differentiate its two usages with conflicting values based on Chinese texts: its high value rhetoric usage (“How dare you……?”) and the default low power (i.e. “You have the courage/resolution to……”) usage share the same form. Second, according to Ma’s discussion, the modal verb 要 *yào* has two meanings “must” and “wish” belonging to the category Necessity Verbs and the category Inclination (Wish) Verbs, which correspond to two different modal values High and Median. Since reliable contextual disambiguation on Google Books’ size data is beyond the scope of the current study, we do not include this polysemous word in the current study. In total, we examined 19 Chinese modal verbs. Recall that Halliday [29] defines high value modal verbs as those involving the obligation, responsibility, and necessity of the target event; median value modal verbs as those expressing the speaker’s will to the target event; and low value modal verbs as those used to express the possibility or ability of the target event. In other words, high value modal verbs commit *the addressee*, median value modal verbs commit *the speaker*, and low value modal verbs commit *neither*. (Note that other components of a sentence also contribute to interpersonal relations. Take the median value modal verb “want” of inclination as an example. “I want to” clearly indicates and commits the inclination of the speaker. On the other hand, although “you want to” is about the inclination of the addressee, it only commits the speaker’s judgment on the inclination. This is the operational criteria we used to assign the values to the modal verbs.) As the previously published list of modal values was all based on intuitive judgment, we revisited them and applied the tripartite levels of commitment criteria we revised to assign the range of modal verbs to modality values, as shown in Table 4.

**Table 4:** The values of Chinese modal verbs.

Value	Words
High	必须 *bìxū* ‘must,’ 当 *dāng* ‘should,’ 得 *děi ‘have to*,*’* 该 *gāi* ‘ought to,’ 须 *xū* ‘must,’ 应 *yīng* ‘should,’ 应当 *yīngdāng* ‘should,’ 应该 *yīnggāi* ‘ought to’, 总得 *zǒngděi* ‘be bound to’
Median	肯 *kěn* ‘be ready to,’ 乐意 *lèyì* ‘be pleased to,’ 想 *xiǎng* ‘want to,’ 愿 *yuàn* ‘be willing,’ 愿意 *yuànyì* ‘be willing’
Low	会 *huì* ‘be likely to,’ 可能 *kěnéng* ‘be possible,’ 可以 *kěyǐ* ‘may,’ 能 *néng* ‘be able to,’ 能够 *nénggòu* ‘be able to’

## Resolving design issues of corpus-based diachronic studies: A calibration study on English modal verbs

Two prominent corpus-based studies on diachronic changes arrived at contradicting conclusions. Leech [1], relying on the well-balanced though relatively small Brown family corpora, concluded that usage of modal verbs was declining. Millar [11] used the unbalanced TIME corpus of 100 million words and concluded that the trend should be increasing instead. Millar [11] pointed out that the point-to-point design of Leech [1] did not provide direct evidence for the diachronic trend and simultaneously argued for the advantage of a bigger corpus. In response, Leech [10] pointed out the risk of using an unbalanced corpus from a single source. In the current study, we try to reconcile the seemingly diagonal criteria of balance vs. size by choosing the largest available corpus that comprehensively covers a variety of genres and topics. An at-a-glance comparison of these three corpora is given in Table 5.

**Table 5:** Comparison of data scale and categories of three corpora.

Corpus	Data Scale	Data Type
The Brown Corpus used by Leech [1]	1 million words in total less than 20,000 words each category	“balanced” samples from 15 written categories
TIME Corpus used by Millar [11]	over 100 million words	sampled from TIME magazine
2012 version of the Chinese part of the Google Books corpus [42]	26,859,461,025 words	sampled from published books of a wide range of topics and genres

The theoretical preference of a balanced corpus is based on the desire for a corpus to be a faithful sample of the totality of language uses of that particular time [49]. To build a robust guideline for the composition of a balanced corpus, the Georgetown team did a comprehensive survey of the reading behavior of college-aged students in the 1960s and set up the criteria for distribution of different genres to be included in Standard Sample of Present-Day American English (the Brown Corpus [49]). This definition of a “balanced corpus” is commonly used in corpus linguistics, especially for English corpus linguistics. Corpora that are balanced in this way include Brown Corpus and Brown Family corpora, such as the Lancaster-Oslo-Bergen Corpus. However, later, other authors of other balanced corpora found that the topic and genre distribution stipulated by the Brown corpus is no longer valid for the specific period of time and/or the language their corpora represents. Hence, they either conducted an updated survey for a different set of balanced distribution (e.g. British National Corpus [50]); or adopt multi-dimensional criteria to define a balanced corpus, such as the Sinica Corpus [51], which is balanced according to the topic, genre, register, and medium. This divergence underlines the theoretical motivation of balancing a corpus by proportionally extracting data based on the actual distribution of the totality of language uses. Without the support of any survey of the targeted population, directly adopting the same distribution for other times or languages is methodologically questionable, and most likely led to an unbalanced sampling of the target time and language.

Adopting an approach of as much data as possible, such as the web-as-corpus approach [40] and the strategy espoused by Millar [11] faces a different set of challenges. On one hand, other things being equal, it is safe to argue that bigger data is better data. However, how big is big enough or good enough? There is no standard formula to follow. On the other hand, it is also fair to assume that homogenous data from the same source cannot be representative enough of the totality of the complexity of language use. Yet, without knowing the full composition of the data, how can one figure out the distribution of a balanced sample?

Given that different distribution patterns of usage are one of the characteristics of language change, we can be fairly sure that a) the Brown Corpus is fairly balanced (for the 1960s); b) BNC and Sinica Corpus are fairly balanced for the time when their balancing criteria were set. We can also be somewhat confident that Brown family corpora for 1990 or later are not very balanced (as the overall distribution of language use in the 1990s is very different from that of the 1960s). On the other hand. Google Books of the 2012 version are randomly sampling (6%) of all available published work. Statistically speaking, random sampling from a (near-complete) set of data is more likely to be representative than very limited sampling based on criteria set for a different time (such as Brown family corpora). We argue that the Google Books corpus is better for diachronic comparison as the sampling varies according to the total data available from each particular year, instead of some fixed distribution set based on past data. The Google Books corpus has the additional strength of being able to support continuous longitudinal studies, and thus addressing the methodological issues of discontinuous point-to-point direct comparisons in diachronic studies, as pointed out by Millar [11].

In this first study, we propose a sampling approach to address the above challenges. An obvious, although potentially unpractical, a solution is to include the totality of all language uses. Barring that impossible goal, the best alternative is to have a comprehensive subset of the total data that is big enough to be reasonably well distributed. The Google Books corpus, composed of a large number of accessible published works, meets the criteria. In addition, its coverage of time and size suggests that the data used by Leech [1] and Millar [11] are largely subsumed. This last fact is crucial in allowing our study to be directly compared to both previous studies.

In terms of the method used to calculate usage trends of the English modal verbs, we follow Leech [1] and Millar [11] in reporting the per million-word usage frequency of each modal verb. Note that we do continuous longitudinal monitoring by calculating the usage frequencies based on all available data in each decade within the scope of the study. Millar [11] pointed out the methodological issue of Leech’s [1] point-to-point direct comparison of data from two random discontinuous years, yet his method of adding (still discontinuous) data points does not change the fundamental risk of missing important variations from the non-sampled years. Our continuous decade-by-decade end-to-end monitoring in Table 6 addresses the root of the issue and allows us also to cover the two periods of observation from the two previous studies: between the 1960s and the 1990s and from the 1920s to the 2000s.

**Table 6:** a. Total match_count of every decade in the English 2012 version of the Google Books corpus (total match_count refer to the total number of words). b. Relative Frequency of English Modal verbs in the Google Books corpus (per million words).

each decade		1901–1910		1911–1920		1921–1930		1931–1940		1941–1950		1951–1960
Total match_count		13482327877		12598499934		11844331903		11490625153		12118806650		18381802696
each decade		1961–1970		1971–1980		1981–1990		1991–2000		2001–2010		
Total match_count		33695717128		42504009610		56780340959		86607874216		117631602601		
Value	Modal Verbs	1901–1910	1911–1920	1921–1930	1931–1940	1941–1950	1951–1960	1961–1970	1971–1980	1981–1990	1991–2000	2001–2010
Low	can_VERB	1297.25	1360.07	1325.98	1336.85	1473.45	1546.57	1605.45	1711.38	1830.50	1943.43	1986.24
	could_VERB	884.82	842.49	881.73	920.51	916.79	911.91	893.56	875.84	903.55	918.40	1019.36
	may_VERB	1552.02	1626.30	1497.29	1457.48	1525.21	1492.67	1386.90	1329.52	1238.13	1135.01	1063.43
	might_VERB	481.25	433.81	451.44	457.87	427.08	421.81	416.51	391.09	372.70	382.34	422.38
	Total	4215.34	4262.67	4156.44	4172.70	4342.52	4372.96	4302.42	4307.83	4344.88	4379.18	4491.41
Median	will_VERB	1618.70	1703.98	1589.67	1495.33	1580.41	1454.84	1399.94	1410.43	1395.13	1385.79	1470.17
	would_VERB	1598.32	1580.14	1616.32	1642.75	1605.60	1582.31	1572.20	1522.36	1474.60	1439.32	1563.06
	should_VERB	1064.86	1116.81	1042.40	979.75	982.92	921.96	866.82	837.34	781.48	730.73	752.48
	shall_VERB	678.24	642.00	592.10	484.06	470.72	436.14	386.61	309.60	223.73	191.37	228.48
	Total	4960.12	5042.93	4840.49	4601.89	4639.65	4395.25	4225.57	4079.73	3874.94	3747.21	4014.19
High	must_VERB	887.89	930.11	889.43	864.27	865.67	818.47	780.49	708.41	645.07	589.88	589.08
	ought_VERB	112.34	96.40	87.71	73.58	61.21	58.88	55.82	44.52	36.15	32.23	40.12
	need_VERB	89.83	99.66	97.60	100.71	111.88	112.59	110.07	129.17	166.99	223.16	263.76
	Total	1090.05	1126.17	1074.74	1038.56	1038.76	989.94	946.38	882.09	848.21	845.27	892.95
Total relative frequency of English modal verbs		10265.51	10431.78	10071.67	9813.15	10020.93	9758.15	9474.37	9269.66	9068.04	8971.66	9398.55

First, we focus on the discrepancies between the two studies shown earlier in Table 1. Note that Millar’s [11] data coverage (1923–2006) starts 38 years before Leech’s [1] (1961) and ends 15 years after (1992). Hence, any results from Millar sharing directionality with Leech and with a higher percentage should be considered as supporting Leech’s conclusion in Table 1. These include *must*, *shall*, and *ought (to)*. As Leech’s study showed a consistent negative growth trend in usage, the seven modal verbs showing a positive trend in Millar’s research should be the focus. Note that since Millar’s study covers a longer period than Leech’s, a small swing in either direction could result from the additional time. Based on these considerations, we treat small swings in direction, especially those with relatively small gaps, as inconclusive. These include *should*, *will* and *would*. The inconsistencies between the two studies for these modal verbs are 15.35% 14.6% and 8.65% respectively. In addition, they show very weak tendency in one direction in Millar’s study (*should* 2.70%, *will* 7.70% and *would* 0.10%), and could reflect either almost no change or at most moderate increase. Four other modal verbs share same tendencies of decrease that differ only in scale (e.g. *ought (to)*, *must*, and *shall*). We also treat them as having compatible results in two studies. Thus, the four modal verbs that yielded clearly identifiable incompatible results with different directions of change from the two studies are *can*, *could*, *may* and *might* with the differences at 113.05%, 105.20%, 84.06% and 25.5% respectively.

**Fig 1:**
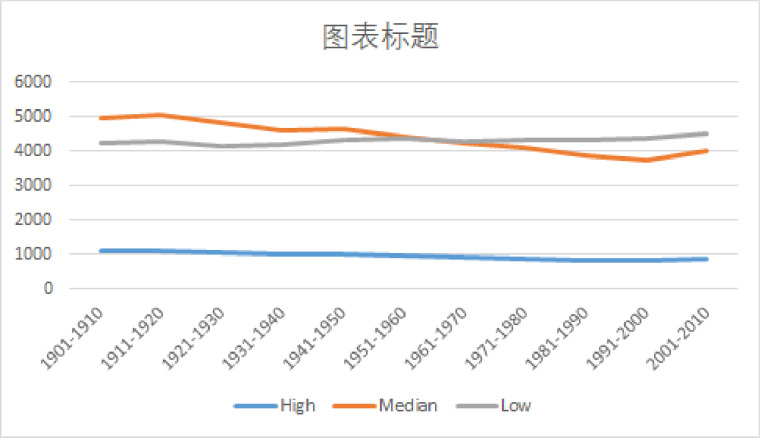
Usage trends of high, median, and low value English modal verbs.

Our longitudinal and continuous monitoring approach produces an informative and detailed overall picture that potentially accounts for the discrepancies between Leech [1] and Millar [11]. Table 6B and Fig 1 provide a clear general pattern of change when modal verbs are classified according to their high-median-low values. According to Leech [1], overall modal verbs are declining, The Google Books corpus confirms this trend during 1911–1940 and 1941–2000 respectively. But there is an increase in the 1940s and they have been rising during 2001–2010. It remains to be seen if they will continue their previous trends or change directions and requires more data and longer observations for the new directions to settle. We can also attest to multiple shorter-term fluctuations, confirming the methodological concerns against simple time point to time point comparisons. The existence of fluctuations also showed that the differences between the two studies are partly due to their different coverage of duration. Direct comparisons with these two studies are given in Tables 7 and 8.

**Table 7:** a. Comparison between Brown-Frown and the Google Books corpus (sorted by trends). b. Comparison between Brown-Frown and the Google Books corpus (sorted by modal verbs).

a										
Brown-Frown					the Google Books corpus					
word	Leech change in diff % (1961~1991–2) of British English	Leech change in diff % (1961~1991–2) of American English	**Leech change average**	Change trend	word	**1960s~1990s**	1961~1991	1961~1992	**1961~1991–2 average**	Change trend
can	2.20%	-1.50%	**0.35%**	slight increase	need	**102.74%**	70.66%	71.97%	**71.32%**	large increase
could	2.40%	-6.80%	**-2.20%**	small decrease	can	**21.05%**	18.55%	20.79%	**19.67%**	
will	-2.70%	-11.10%	**-6.90%**		could	**2.78%**	3.49%	0.37%	**1.93%**	slight increase
would	-11.00%	-6.10%	**-8.55%**		will	**-1.01%**	-2.70%	-3.11%	**-2.91%**	small decrease
might	-15.10%	-4.50%	**-9.80%**		would	**-8.45%**	-7.51%	-9.89%	**-8.70%**	
should	-11.80%	-13.50%	**-12.65%**		might	**-8.20%**	-10.15%	-12.01%	**-11.08%**	
may	-17.40%	-32.40%	**-24.90%**	large decrease	should	**-15.70%**	-14.83%	-15.77%	**-15.30%**	
need (n’t)	-40.20%	-12.50%	**-26.35%**		may	**-18.61%**	-20.53%	-19.42%	**-19.97%**	large decrease
must	-29.00%	-34.40%	**-31.70%**		must	**-24.42%**	-23.30%	-24.23%	**-23.76%**	
ought (to)	-44.20%	-30.00%	**-37.10%**		ought	**-42.26%**	-42.03%	-42.93%	**-42.48%**	
shall	-43.70%	-43.80%	**-43.75%**		shall	**-50.50%**	-47.28%	-49.10%	**-48.19%**	
b										
Brown-Frown				the Google Books corpus						
word	Leech change(1961~1991–2) of British English	Leech change (1961~1991–2) of American English	**Leech change average**	word	**1960s~1990s**	1961~1991	1961~1992		**1961~1991–2 average**	
can	2.20%	-1.50%	**0.35%**	can	**21.05%**	18.55%	20.79%		**19.67%**	
could	2.40%	-6.80%	**-2.20%**	could	**2.78%**	3.49%	0.37%		**1.93%**	
may	-17.40%	-32.40%	**-24.90%**	may	**-18.61%**	-20.53%	-19.42%		**-19.97%**	
might	-15.10%	-4.50%	**-9.80%**	might	**-8.20%**	-10.15%	-12.01%		**-11.08%**	
must	-29.00%	-34.40%	**-31.70%**	must	**-24.42%**	-23.30%	-24.23%		**-23.76%**	
need (n’t)	-40.20%	-12.50%	**-26.35%**	need	**102.74%**	70.66%	71.97%		**71.32%**	
ought (to)	-44.20%	-30.00%	**-37.10%**	ought	**-42.26%**	-42.03%	-42.93%		**-42.48%**	
shall	-43.70%	-43.80%	**-43.75%**	shall	**-50.50%**	-47.28%	-49.10%		**-48.19%**	
should	-11.80%	-13.50%	**-12.65%**	should	**-15.70%**	-14.83%	-15.77%		**-15.30%**	
will	-2.70%	-11.10%	**-6.90%**	will	**-1.01%**	-2.70%	-3.11%		**-2.91%**	
would	-11.00%	-6.10%	**-8.55%**	would	**-8.45%**	-7.51%	-9.89%		**-8.70%**	

**Table 8:** a. Comparison results between TIME and the Google Books corpus from 1920s to 2000s (sorted by trends). b. Comparison results between TIME and the Google Books corpus from 1920s to 2000s (sorted by modal verbs).

TIME corpus 1920s-2000s			the Google Books corpus 1920s-2000s	
large increase	may	59.7%	can	49.79%
	can	113.4%	could	15.61%
	could	103.0%	would	-3.30%
small increase	will	7.7%	might	-6.44%
fluctuation	might	15.7%	will	-7.52%
	should	2.7%	should	-27.81%
	would	0.1%	may	-28.98%
large decrease	ought	-44.9%	ought	-54.26%
	must	-48.4%	must	-33.77%
	shall	-95.6%	shall	-61.41%

Modal Verbs	TIME	the Google Books corpus
	1920s-2000s	1920s-2000s
can	113.40%	49.79%
could	103.00%	15.61%
may	59.70%	-28.98%
might	15.70%	-6.44%
shall	-95.60%	-61.41%
should	2.70%	-27.81%
will	7.70%	-7.52%
would	0.10%	-3.30%
must	-48.40%	-33.77%
ought	-44.90%	-54.26%

Table 7 shows that, unlike the results from the Brown family corpora based study [1], the use of modal verbs *can* and *could* increased between 1961 and 1991–2 based on our study, consistent with Millar [11]. The other eight modal verbs showed roughly comparable trends. Looking at Table 7, it is also shown that Leech’s [1] observation of strong overall falling trends is in fact a feature of the particular period he chose for his study (i.e. 1961~1991–2) and does not necessarily apply beyond that period. Table 9 shows the change trends of English modal verbs. For example, *can* was increasing from 1920s to 2000s; *could* was increasing from 1970s to 2000s; *need* was increasing from 1960s to 2000s.

**Table 9:** a. Change trends of English modal verbs. b. Change trends of English modal verbs with three or more continuous decades.

a		
Modal Verbs	Decrease	Increase
can	1910s~1920s	1900s~1910s, 1920s~2000s
could	1900s~1910s, 1930s~1970s	1910s~1930s, 1970s~2000s
may	1910s~1930s, 1940s~2000s	1900s~1910s, 1930s~1940s
might	1900s~1910s, 1930s~1980s	1910s~1930s, 1980s~2000s
will	1910s~1930s, 1940s~1960s, 1970s~1990s	1900s~1910s, 1960s~1970s, 1990s~2000s
would	1900s~1910s, 1930s~1990s	1910s~1930s, 1990s~2000s
should	1910s~1930s, 1940s~1990s	1900s~1910s, 1930s~1940s,1990s~2000s
shall	1900s~1990s	1990s~2000s
must	1910s~1930s, 1940s~2000s	1900s~1910s, 1930s~1940s
ought	1900s~1990s	1990s~2000s
need	1910s~1920s, 1950s~1960s	1900s~1910s, 1920s~1950s, 1960s~2000s
Total of low value modal verbs	1910s~1920s, 1950s~1960s	1900s~1910s, 1920s~1950s, 1960s~2000s
Total of median value modal verbs	1910s~1930s 1940s~1990s	1900s~1910s, 1930s~1940s, 1990s~2000s
Total of high value modal verbs	1910s~1930s, 1940s~1990s	1900s~1910s, 1990s~2000s
Total	1910s~1930s, 1940s~1990s	1900s~1910s, 1930s~1940s, 1990s~2000s
b		
Modal Verbs	Decrease	Increase
can	/	1920s~2000s
could	1930s~1970s	1970s~2000s
may	1940s~2000s	/
might	1930s~1980s	1980s~2000s
will	1940s~1960s, 1970s~1990s	/
would	1930s~1990s	/
should	1910s~1930s, 1940s~1990s	/
shall	1900s~1990s	
must	1910s~1930s,1940s~2000s	/
ought	1900s~1990s	/
need	/	1920s~1950s, 1960s~2000s
Total of low value modal verbs	/	1920s~1950s,1960s~2000s
Total of median value modal verbs	1910s~1930s, 1940s~1990s	/
Total of high value modal verbs	1940s~1990s	/
Total	1910s~1930s,1940s~1990s	/

Table 8 compares our results with those from Millar [11], which reported increases of usages of seven modal verbs in varying degrees between the 1920s and 2000s: *can*, *could*, *may*, *might*, *should*, *will* and *would*. This study showed increasing trends only for *can* and *could*. Given the larger size and more balanced nature of our corpus, that these two modal verbs also showed modest gains in British English of Leech’s study [1], and that they are low value modal verbs with similar meanings, it is reasonable to conclude that our result is the more reliable one and that the other increases reported by Millar [11] are corpus specific.

Our end-to-end longitudinal study has provided more detailed information and has captured more nuanced usage trends and changes over different time periods. Most crucially, we are also able to reconcile some of the discrepancies and provide possible explanations for the remaining inconsistencies between the two previous studies by Leech [1] and Millar [11]. Through the consideration of decade-to-decade trends in Tables 6 and 9, this study has shown that most modal verbs were decreasing. Based on the value assignments of modal verbs [29], *would* is assigned median value in the literature but could be assigned a low value according to the level of commitment interpretation we gave earlier in this paper. In other words, the English language showed a clear trend of moving from modal verbs that require high-low power relations and demand high levels of obligations. The overall decrease of high and median value modal verbs is partially compensated by the increasing usage of low value modal verbs. In other words, the written discourse is moving from high value demands of obligations to low value non-committing peer perspectives. Following Leech’s [1] hypothesis that the language use trends reflect the societal move to more open and equal interaction, our dichotomy of low value versus high/median modals should provide an even more nuanced account. In addition, in terms of functional compensation of linguistic changes and empowering the speakers, the increasing use of low value modal verbs is also predicted. Among the low value modal verbs, *can* and *could* can be argued to be more empowering than *may* and *might* according to their meaning. The ability meaning of *can* and *could* implies that addressees have the power to act volitionally. On the other hand, *may* and *might* have basic meaning of giving permissions and are often used in hypothetical and hedging contexts, thus leaving the volitional power of the addresses unspecified. In other words, our data is not only more reliable but also better aligned with theoretical predictions.

## The main study on Mandarin: Data preparation

We apply the same longitudinal continuous sampling approach to study Mandarin Chinese modal verbs, having established the merit of this approach in comparison to previous studies of English. Our data is extracted from the 2012 version of Chinese (simplified) Google Ngram (https://storage.googleapis.com/books/ngrams/books/datasetsv3.html), which converts the full content of the Google Books corpus into an Ngram database. An obvious technical challenge in this approach is how to define the beginning and end of a sampling period. Fortunately, most of the critical events covered in this study occur in the first or last year of the numeric decades (e.g. 1921, 1949, and 1960). Hence, we took the numeric decades as the units of sampling, assuming the immediately following years (i.e. 1920s, 1950s, and 1960s) as the duration of change influenced by the events. Future studies could adjust the beginning and ending years of per-decade sampling to focus on events occurring in mid-decade years.

In this study, modal verbs are extracted as monograms and with “VERB” as the PoS tag (i.e. “*word*_VERB”). In addition, we also extract the “total_counts” of sub-corpora in order to normalize the reported use frequency as the number of usages per million words. The downloaded total_counts data is presented in a text file. Every set contains four parameters separated by commas and the TAB keys are used to separate each set of data. The specific parameters are year, match_count, page_count, volume_count. We used the total match_counts data in Table 10A from the corpus to calculate the relative usage frequency of each modal verb (per million words). The calculation formula of relative frequency is as follows, with the result in Table 10B.

**Table 10:** a. Total match_count of each decade in the Chinese 2012 version of the Google Books corpus. b. Total number of Chinese modal verbs from 1901 to 2009.

Decade	1901–1910	1911–1920	1921–1930	1931–1940	1941–1950	1951–1960
Total match_count	923357	903024	3216692	7673962	10817251	233220966
Decade	1961–1970	1971–1980	1981–1990	1991–2000	2001–2009	/
Total match_count	193996112	887460400	7202341748	9626166540	8681142274	/
Value	Modal Verbs		match_count		Relative frequency	Number of Books
High	必须 *bìxū* ‘must’		21363340		6106.13	259337
	当 *dāng* ‘should’		3754551		1198.69	247461
	得 *děi* ‘have to’		6355319		2911.64	270189
	该 *gāi* ‘ought to’		2087038		551.71	219359
	须 *xū* ‘must’		2046672		805.38	206860
	应 *yīng* ‘should’		20329535		7181.25	268742
	应当 *yīngdāng* ‘should’		9531099		2590.04	212113
	应该 *yīnggāi* ‘ought to’		10439667		2626.45	234965
	总得 *zǒngděi* ‘be bound to’		205		0.05	203
Median	肯 *kěn* ‘be ready to		371921		186.36	124389
	乐意 *lèyì* ‘be pleased to’		60397		16.73	39710
	想 *xiǎng* ‘want to’		9866200		3834.54	250640
	愿 *yuàn* ‘be willing’		1342214		360.39	192560
	愿意 *yuànyì* ‘be willing’		1992281		577.36	189427
Low	会 *huì* ‘be likely to’		37336729		11981.42	277232
	可能 *kěnéng* ‘be possible’		15794920		5295.11	264040
	可以 *kěyǐ* ‘may’		44662710		19215.79	284869
	能 *néng* ‘can’		28050209		9474.26	285046
	能够 *nénggòu* ‘be able to’		8430621		3139.02	246439

Relative Frequency = (year_counts / total match_counts) * 1,000,000

Note that the use of normalized relative frequency is necessary given the nature of the Google Books corpus. There are no sampling size constraints. The corpus size is determined by the “as much as possible” sampling guideline instead of a fixed total and distribution for each sample point. Hence absolute frequency depends more on the availability of data and it is not directly related to the actual usage. However, we did check unusual peaks and troughs to ensure that significant trends were not missed. Distributions in Table 10 support the hypothesis that modal verbs were frequently used and found in all types of texts, hence a good feature for monitoring linguistic changes.

## Results and analysis

We introduce in this section the significant changes in usages of Chinese modal verbs, focusing on general trends shared by modal verbs of the same modality value.

### Relative frequency of each modal verb in every ten years

Recall that each decade is the basic unit for our continuous longitudinal comparison monitoring study, as shown in

Table 11. Each of these modals that are marked as “VERB” in google ngram was extracted. In each decade in the past 109 years, low value modal verbs were used most often, and median value modal verbs were used the least. Among the high value Chinese modal verbs, the most commonly used modal verb is 应 *yīng* ‘should’. We will account for its very prominent peak in the 1940s in the following discussion section. The least used is 总得 *zǒngděi* ‘be bound to.’ Among the median value modal verbs, 想 *xiǎng* ‘want to’ is used most often and 乐意 *lèyì* ‘be pleased to’ is the least used. Among “low” value modal verbs, the most used is 可以 *kěyǐ* ‘may’ and the least used is 能够 *nénggòu* ‘be able to.’ And among all modal verbs, the three most frequently used ones are 可以 *kěyǐ* ‘may,’ 会 *huì* ‘will (future commitment),’ and 能 *néng* ‘can,’ all low value modal verbs.

**Table 11:** Relative frequency of Chinese modal verbs in the Google Books corpus (per million words).

Value	Modal Verbs	1901–1910	1911–1920	1921–1930	1931–1940	1941–1950	1951–1960	1961–1970	1971–1980	1981–1990	1991–2000	2001–2009	Total
High	必须 *bìxū* ‘must’	171.11	52.05	156.06	254.24	880.91	743.19	644.28	797.18	895.82	818.64	692.65	6106.13
	当 *dāng* ‘should’	25.99	16.61	64.66	58.64	81.91	177.67	181.29	175.11	150.93	133.08	132.8	1198.69
	得 *děi* ‘have to’	98.55	118.49	313.68	361.74	255.33	368.4	372.77	321.93	262.97	222.37	215.41	2911.64
	该 *gāi* ‘ought to’	22.74	32.11	25.49	28.93	42.06	38.65	43.84	83.54	76.03	76.78	81.54	551.71
	须 *xū* ‘must’	27.08	27.68	75.85	81.18	153.55	73.94	68.07	68.73	80.67	86.08	62.55	805.38
	应 *yīng* ‘should’	361.72	193.79	339.79	634.48	1626.2	730.32	506.77	493.2	762.14	849.91	682.93	7181.25
	应当 *yīngdāng* ‘should’	20.58	13.29	32.95	51.73	276.41	545.88	316.52	268.47	307.39	335.07	421.75	2590.04
	应该 *yīnggāi* ‘ought to’	68.23	38.76	92.33	142.69	400.75	63.85	163.66	467.08	450.92	335.25	402.93	2626.45
	总得 *zǒngděi* ‘be bound to’	0	0	0	0	0	0	0.01	0.01	0.01	0.01	0.01	0.05
	Total	796	492.78	1100.81	1613.63	3717.12	2741.9	2297.21	2675.25	2986.88	2857.19	2692.57	23971.34
Median	肯 *kěn* ‘be ready to	8.66	4.43	24.25	22.54	12.2	24.22	24.92	24.74	16.14	12.94	11.32	186.36
	乐意 *lèyì* ‘be pleased to’	1.08	1.11	0	1.17	0.65	2.14	1.48	2.29	2.4	2.14	2.27	16.73
	想 *xiǎng* ‘want to’	66.06	84.16	314.92	327.73	223.81	578.84	596.33	564.56	383.38	334.28	360.47	3834.54
	愿 *yuàn* ‘be willing’	10.83	8.86	22.38	30.49	24.96	28.02	27.9	55.95	53.59	51.19	46.22	360.39
	愿意 *yuànyì* ‘be willing’	25.99	8.86	21.76	42.87	35.78	66.96	63.61	89.94	71.31	68.24	82.04	577.36
	Total	112.62	107.42	383.31	424.8	297.4	700.18	714.24	737.48	526.82	468.79	502.32	4975.38
Low	会 *huì* ‘be likely to’	531.76	387.59	668.08	645.56	887.56	1679.84	1565.66	1456.79	1362.6	1316.94	1479.04	11981.42
	可能 *kěnéng* ‘be possible’	96.39	47.62	206.42	367.09	516.58	902.15	796.22	604.55	593.29	528.48	636.32	5295.11
	可以 *kěyǐ* ‘may’	552.33	485.04	2588.37	2152.21	1695.26	2742.06	2260.02	1778.88	1695.26	1477.69	1788.67	19215.79
	能 *néng* ‘can’	308.66	244.73	757.61	910.87	685.48	1130.44	1129.64	1168.49	1129.21	993.15	1015.98	9474.26
	能够 *nénggòu* ‘be able to’	40.07	26.58	202.38	159.89	235	656.81	529.35	360.58	307.64	260.36	360.36	3139.02
	Total	1529.21	1191.56	4422.86	4235.62	4019.88	7111.3	6280.89	5369.29	5088	4576.62	5280.37	49105.6
Total relative frequency of Chinese modal verbs		2437.83	1791.76	5906.98	6274.05	8034.4	10553.38	9292.34	8782.02	8601.7	7902.6	8475.26	78052.32

### Usage trends

**Fig 2:**
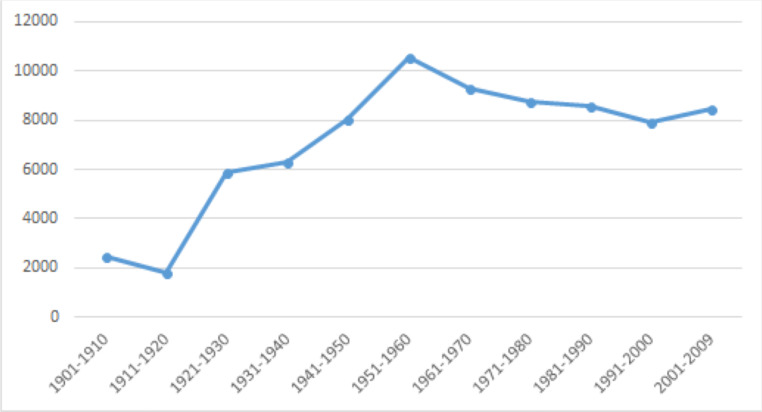
Usage trends of all Chinese modal verbs.

Fig 2 is based on the relative frequency of all modal verbs in each decade of Table 11. It shows that there are two spikes of total modal verb usages: in the 1920s and 1950s. Although each modal verb shows different patterns of changes, the spikes are followed by either a slow increasing pattern (1930s) or a decreasing pattern (1960s on). In what follows, we will first look at the trends of low, median, and high value modal verbs, respectively, before concluding with an overall view of how these three types of modal verbs interact.

**Fig 3:**
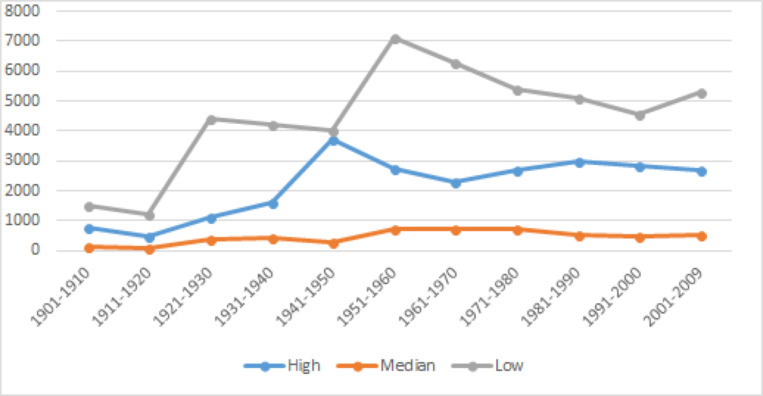
Usage trends of high, median and low value Chinese modal verbs.

To explore the correlation between modality values and the usage trends of Chinese modal verbs, results are presented in Fig 3 for the relative frequency of all “high,” “median,” and “low” based on Table 11, respectively.

#### Usage trends of high value modal verbs

The overall usage trend of high value Chinese modal verbs in

**Fig 4:**
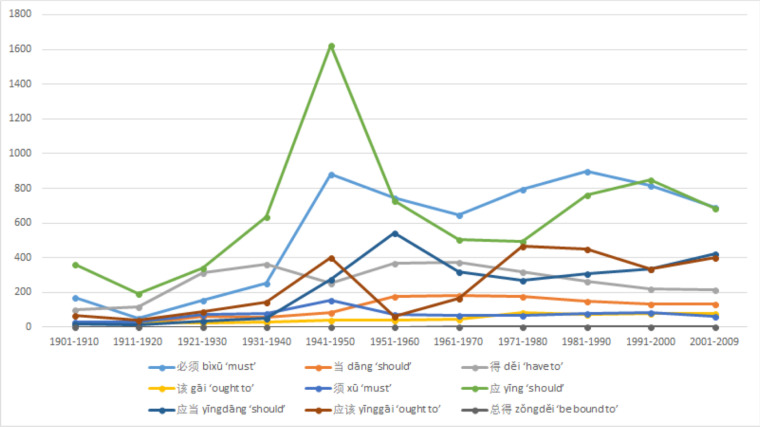
Usage trends of each high value Chinese modal verb.

Fig 3 can be summarized as follows: A single peak in the 1940s, preceded by rising usages and followed by mild fluctuations with modest decreasing tendencies. The drastic peak in the 1940s happened when China was under upheaval. The fight against the Japanese invasion (as part of World War II) is separated only by a short lull before the Civil War between the Chinese Communist Party and the Nationalist Kuomintang that led to the establishment of PRC in 1949. The increased usage (and significant decrease soon after) corresponds with the necessity for collective action and the more authoritarian shift of the society at war. The earlier and more gradual rise in the 1920s coincided with the New Culture Movement. Progressive intellectuals started the New Culture Movement in the 1910s, with the promotion of vernacular writing that would end a two thousand year old tradition of writing only in classical style and being reserved for the educated few. It cultivated the national and international awareness in the May Fourth Movement, and hence in a broader sense 五四运动 *wǔsì yùndòng* ‘May Fourth Movement’ has been commonly used to refer to both the New Culture Movement and the May Fourth Movement. This change encouraged frequent uses of the colloquial modal verbs. This is a trend that should be shared by all modal verbs regardless of value differences. Another noticeable trend is the overall growing popularity of disyllabic verbs, which is shown in Fig 4. Other than the low-frequency 总得 *zǒngděi* ‘be bound to,’ which is used predominantly as an adverb according to Sinica Corpus [37, 51], three of the four most frequently used high modal verbs are disyllabic: 应该 *yīnggāi* ‘ought to,’ 应当 *yīngdāng* ‘should,’ and 必须 *bìxū* ‘must.’ The other high frequency, high value modal verb is 应 *yīng* ‘should’.

#### Usage trends of median value modal verbs

**Fig 5:**
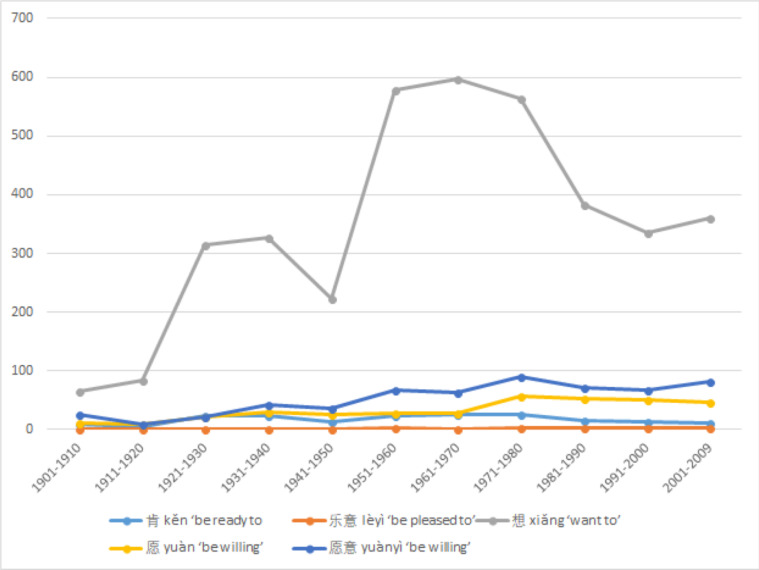
Usage trends of each median value Chinese modal verbs.

The usage of median value modal verbs has fluctuated in the past century at a relatively low level. The only median value modal verb with significant usage change is 想 *xiǎng* ‘to think, to want to’ shown in Fig 5. Although its basic meaning is ‘to think,’ colloquially the modal verb meaning of ‘to want to’ has become dominant in modern Mandarin. This is probably derived from contextual implicature (an expressed thought should be contextually relevant and a salient relevance would share a goal of the speaker in order to achieve it). It is also a popular request verb as it lacks the explicit request form of directing to the addressee. Contrary to high value requirement type modal verbs, the usage of 想 *xiǎng* plunged during the war years of the 1940s and recovered after the founding of the PRC in 1949. The other interesting trend is the recent rise of 愿意 *yuànyì* ‘be willing,’ which is often used to translate ‘I do’ in wedding vows and is also used in pledging. This last trend could be a topic for a different study.

#### Usage trends of low values modal verbs

It is important to note that low value modal verbs are consistently the most dominant and most frequently used type throughout all decades.

**Fig 6:**
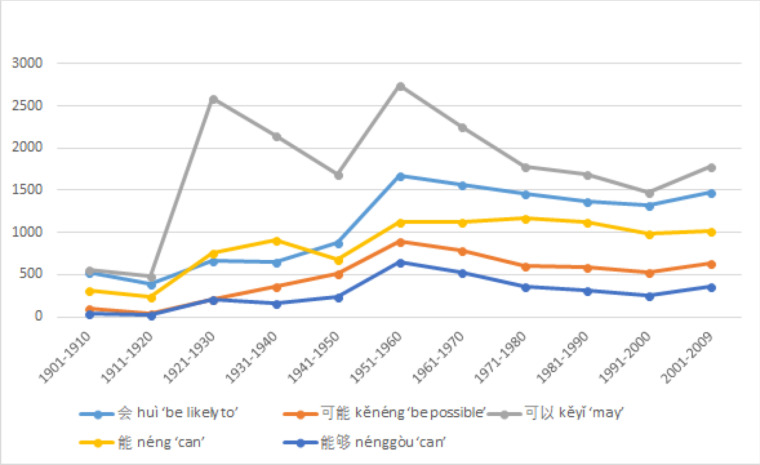
Usage trends of each low value Chinese modal verb.

Fig 3 shows two spikes in total usage of modal verbs in the 1920s and 1950s, and also shows a slight downward trend after the peak. These spikes and the general downward trends after the peak provide strong contextual information that the sudden increase was directly influenced by a significant socio-cultural event. A slight decline is inconsistent with the rising S-curve model typical of replacement language change [52], but could be accounted for if it is the result of external cause whose effect could wear off gradually over time. These coincide with the impact of the New Culture Movement and the founding of PRC. Among them, 可以 *kěyǐ* ‘may’ is the most frequently used word shown in Fig 6, again affirming the disyllabic trend. It should also be noted that all low value modal verbs had peak usage during the 1950s.

Lastly, an overall direct comparison of the usage trends of according values was given earlier in Fig 3. Several important generalizations can be observed. First, the frequency of low value modal verbs is consistently and significantly higher than the other two types. This seems to be an important feature of Mandarin Chinese, as the same does not hold for English, as can be seen in Tables 6 and 10. Throughout the 110 years of observation, the frequencies of total usage and most frequently used modal verbs in English are very similar between low and median value modal verbs, with low value modal verbs slowly becoming more used.

**Fig 7:**
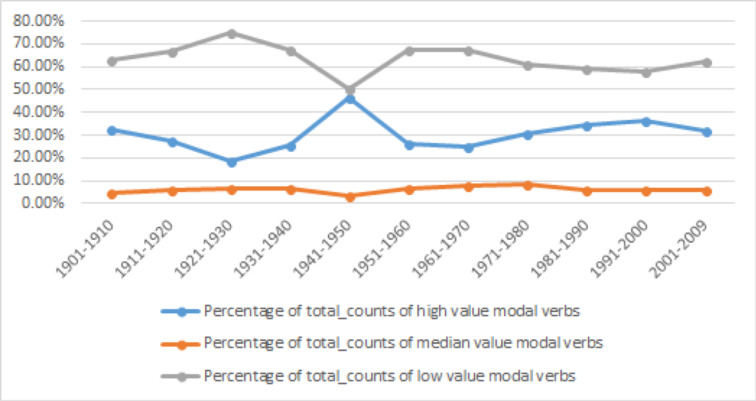
Percentage of Chinese modal verbs of high, median and low values in each decade.

The observation that the distribution of three types of modal verbs according to their values differ significantly in Chinese and English suggests that such distribution could be a very important signature of modal verb usages. In addition, we also note that the concepts of lexical competition and replacement changes in theories of language change/evolution share the premise that competing linguistic units are vying with each other for a shared total of function or information load. Thus, to better envision the interaction of the three different modality values, we calculate the share of each value according to total modal verb usages, as given in Table 12 and Fig 7.

**Table 12:** Percentage of Chinese modal verbs of high, median, and low values in each decade.

Value	Number and Percentage	1901–1910	1911–1920	1921–1930	1931–1940	1941–1950	1951–1960	1961–1970	1971–1980	1981–1990	1991–2000	2001–2009
High	match_counts	735	445	3541	12383	40209	639471	445648	2374169	21512470	27503754	23374601
	Percentage	32.65%	27.50%	18.64%	25.72%	46.27%	25.98%	24.72%	30.46%	34.72%	36.15%	31.77%
Median	match_counts	104	97	1233	3260	3217	163297	138561	654484	3794293	4512724	4360743
	Percentage	4.62%	6.00%	6.49%	6.77%	3.70%	6.63%	7.69%	8.40%	6.12%	5.93%	5.93%
Low	match_counts	1412	1076	14227	32504	43484	1658504	1218468	4765031	36645539	44055349	45839595
	Percentage	62.73%	66.50%	74.88%	67.51%	50.03%	67.38%	67.59%	61.14%	59.15%	57.91%	62.30%
Total match_counts		2251	1618	19001	48147	86910	2461272	1802677	7793684	61952302	76071827	73574939

The quantitative analysis confirms the two generalizations that we observed earlier. First, the category of low value modal verbs is the most dominant, which has a share of 50.03% to 74.88% over the 11 decades. That is, in any given time low value modal verbs would take up at least half of the share of all modal verbs. Second, the share of median value modal verbs is smallest and without significant changes, ranging from 3.70% to 8.4%. In other words, the usage of low and high value modal verbs dominates modal verb usages in Chinese. This polarization of interpersonal power relations is very different from English, where the low and median value modal verbs both dominate. The significance of this contrast deserves in-depth future studies.

Fig 7 shows a very clear picture of competition between high and low value modal verbs in the past century. The changes are mirrored in scale. That is, low value modals will increase their share by roughly the share lost by high value modal verbs, and vice versa. This simple one-on-one competition provides a perfect context for exploring the driving force of the change.

From Fig 2, we can see that the first significant surge of modal verb usages was in the 1920s. From

**Fig 8:**
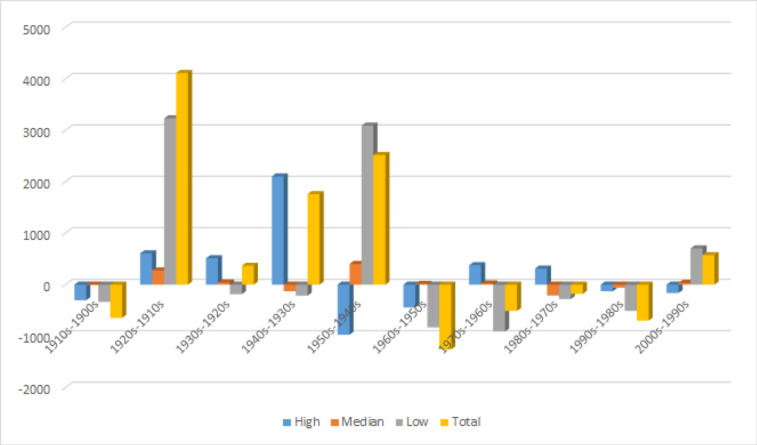
The difference of the relative frequency of Chinese modal verbs between one decade and its previous decade.

Fig 7, we can see that the 1920s also has a significant increase in shares of low value modal verbs, as shown in Table 13. Table 14 and Fig 8 show the difference between one decade and its previous decade.

**Table 13:** Relative frequency of Chinese modal verbs in each decade.

Relative Frequency	1901–1910	1911–1920	1921–1930	1931–1940	1941–1950	1951–1960	1961–1970	1971–1980	1981–1990	1991–2000	2001–2009
High	796	492.78	1100.81	1613.63	3717.12	2741.9	2297.21	2675.25	2986.88	2857.19	2692.57
Median	112.62	107.42	383.31	424.8	297.4	700.18	714.24	737.48	526.82	468.79	502.32
Low	1529.21	1191.56	4422.86	4235.62	4019.88	7111.3	6280.89	5369.29	5088	4576.62	5280.37
Total	2437.83	1791.76	5906.98	6274.05	8034.4	10553.38	9292.34	8782.02	8601.7	7902.6	8475.26

**Table 14:** The difference of the relative frequency of Chinese modal verbs between one decade and its previous decade.

Value	1910s-1900s	1920s-1910s	1930s-1920s	1940s-1930s	1950s-1940s	1960s-1950s	1970s-1960s	1980s-1970s	1990s-1980s	2000s-1990s
High	-303.22	608.03	512.82	2103.49	-975.22	-444.69	378.04	311.63	-129.69	-164.62
Median	-5.2	275.89	41.49	-127.4	402.78	14.06	23.24	-210.66	-58.03	33.53
Low	-337.65	3231.3	-187.24	-215.74	3091.42	-830.41	-911.6	-281.29	-511.38	703.75
Total	-646.07	4115.22	367.07	1760.35	2518.98	-1261.04	-510.32	-180.32	-699.1	572.66

The increase in the usage of all modal verbs in the 1920s (4115.22 per million) can be shown to come mostly from low value modal verbs (3231.3 per million). We can also verify with Table 15 that high and median modal verbs only showed a modest gain in frequency between the 1910s and the 1920s compared with low value modal verbs. Furthermore, we can show that a single low value modal verb 可以 *kěyǐ* ‘may’ contributed most to the increase, and even more than all high and median modal verbs combined. 16 out of the 17 modal verbs that have data during this period increases over 70%, with 能够 *nénggòu* ‘be able to’ having the highest percentage increase at 661.40%.

**Table 15:** The change between 1920s and 1910s.

Value	Modal Verbs	1911–1920	1921–1930	1920s-1910s	Increased Percentage
High	必须 *bìxū* ‘must’	52.05	156.06	104.01	199.83%
	当 *dāng* ‘should’	16.61	64.66	48.05	289.28%
	得 *děi* ‘have to’	118.49	313.68	195.19	164.73%
	该 *gāi* ‘ought to’	32.11	25.49	-6.62	-20.62%
	须 *xū* ‘must’	27.68	75.85	48.17	174.02%
	应 *yīng* ‘should’	193.79	339.79	146	75.34%
	应当 *yīngdāng* ‘should’	13.29	32.95	19.66	147.93%
	应该 *yīnggāi* ‘ought to’	38.76	92.33	53.57	138.21%
	总得 *zǒngděi* ‘be bound to’	0	0	0	0
	Total	492.78	1100.81	608.03	123.39%
Median	肯 *kěn* ‘be ready to	4.43	24.25	19.82	447.40%
	乐意 *lèyì* ‘be pleased to’	1.11	0	0	0
	想 *xiǎng* ‘want to’	84.16	314.92	230.76	274.19%
	愿 *yuàn* ‘be willing’	8.86	22.38	13.52	152.60%
	愿意 *yuànyì* ‘be willing’	8.86	21.76	12.9	145.60%
	Total	107.42	383.31	275.89	256.83%
Low	会 *huì* ‘be likely to’	387.59	668.08	280.49	72.37%
	可能 *kěnéng* ‘be possible’	47.62	206.42	158.8	333.47%
	可以 *kěyǐ* ‘may’	485.04	2588.37	2103.33	433.64%
	能 *néng* ‘can’	244.73	757.61	512.88	209.57%
	能够 *nénggòu* ‘be able to’	26.58	202.38	175.8	661.40%
	Total	1191.56	4422.86	3231.3	271.18%
Total		1791.76	5906.98	4115.22	229.67%

The second significant change for the overall usage of modals happened in the 1940s, where the frequency of modal verbs increased by 1760.35 per million, with high value modal verbs increased by 2103.49 per million, higher than the total increase; in fact, the usage of median and low value modal verbs slightly decreased by 127.4 and 215.74 per million respectively. This is the reason why the overall increase was not as prominent as in the 1920s. Table 12 shows that the share of high value modal verbs peaked, at the expense of the decrease in the share of low and median value modals. We surmise that the change in the 1940s was driven by and related to the increase uses of high value modal verbs. The high value modal verb应 *yīng* ‘should’ made the most contribution to the change with a frequency increase of 991.72 per million as shown in Table 16. Seven out of the eight modal verbs increased over 39%, with 应当 *yīngdāng* ‘should’ having the highest increased percentage at 434.33%.

**Table 16:** The change between 1940s and 1930s.

Value	Modal Verbs	1931–1940	1941–1950	1940s-1930s	Increased Percentage
High	必须 *bìxū* ‘must’	254.24	880.91	626.67	246.49%
	当 *dāng* ‘should’	58.64	81.91	23.27	39.68%
	得 *děi* ‘have to’	361.74	255.33	-106.41	-29.42%
	该 *gāi* ‘ought to’	28.93	42.06	13.13	45.39%
	须 *xū* ‘must’	81.18	153.55	72.37	89.15%
	应 *yīng* ‘should’	634.48	1626.2	991.72	156.30%
	应当 *yīngdāng* ‘should’	51.73	276.41	224.68	434.33%
	应该 *yīnggāi* ‘ought to’	142.69	400.75	258.06	180.85%
	总得 *zǒngděi* ‘be bound to’	0	0	0	0
	Total	1613.63	3717.12	2103.49	130.36%

The final major change happened in the 1950s when total frequencies increased by 2518.98 per million. Similar to the 1920s, low value modal verbs contributed most to the change, by 3091.41 per million. Similar to high value modal verbs in the 1940s, the increased use of low value modal verbs is higher than the total increase. Unlike in the 1940s, the loss came only from high value modals, as median value modal verbs also increased. High value modal verbs losing a significant share that was gained mostly by low value modal verbs. 可以 *kěyǐ* ‘may’ has the largest increase of 1046.8 per million as shown in Table 17. All low value modal verbs increased over 60%, with 能够 *nénggòu* ‘be able to’ increasing significantly at 179.49%.

**Table 17:** The change between 1950s and 1940s.

Value	Modal Verbs	1941–1950	1951–1960	1950s-1940s	increased percentage
Low	会 *huì* ‘be likely to’	887.56	1679.84	792.28	89.26%
	可能 *kěnéng* ‘be possible’	516.58	902.15	385.57	74.64%
	可以 *kěyǐ* ‘may’	1695.26	2742.06	1046.8	61.75%
	能 *néng* ‘can’	685.48	1130.44	444.96	64.91%
	能够 *nénggòu* ‘be able to’	235	656.81	421.81	179.49%
	Total	4019.88	7111.3	3091.42	76.90%

There are no other significant overall or share changes in other decades, we will return to a possible account of the generalizations observed in this section later in the discussion section.

## Discussion

Our end-to-end longitudinal study of modal verbs usages in Mandarin Chinese leads to several interesting findings:

- The distribution as well as competition patterns of modal verbs according to three levels of value modality show language specific differences.
- The changes in modal verb usages in Mandarin in the past 109 years can be generalized as a competition between high and low value modal verbs. These two types of modal verbs mirror their changes in terms of their shares in total modal verb usages.
- Overall, low value modal verbs dominate modal verb usages in Mandarin Chinese in the past century, occasionally challenged by high value modal verbs, with median value modal verbs being used least frequently.
- Significant changes are typically driven by a single modal verb, either low or high value modal verbs.

The competition model could be understood in terms of total information load, similar to well-documented replacement changes in historical linguistics. That is, taking the information load and function of all modal verbs in total, the three types as defined by values are competing against each other to represent the information content. Reduced load for one type will be replaced by other types. A significant feature observed of the competition of modal verbs in the past 109 years is that only two of the three types, low and high values, are in active competition. In addition, instead of the sigmoid snowball effect of replacement, the high and low modal verbs engage in a tug-of-war competition. The familiar replacement model does not account for the polarity anchored competition without the active involvement of median value modal verbs. Nor does it account for the motivation or for the changes or why the back-and-forth changes happened instead of the well-attested sigmoid function of accumulative replacement changes.

To account for the above questions, it is necessary to explicate again the relational differences among the three values of modality. As defined by Halliday and Hasan [31], among others, modality marks interpersonal relations, especially between the speaker and the addressee. The high, median and low value is defined according to the strength of the implicated obligation. The high value modal verbs, such as *must* in English, marked the necessity of the event. They are typically used in a high-low power relation that commits all addressees (the speaker included) to a position. The median value modal verbs, such as *will* in English, mark the inclination of the event. They are typically used in a position with moderate commitment. The low value modal verbs, such as *can* in English, mark the potential of the event. As such, the speaker makes no clear commitment to the happening of the event, thus transferring the decision to commit to the addressee. The low value modal verbs are empowering in the sense of allowing the addressee to make the event happen (or not). It is crucial to note that in terms of interpersonal interaction and collective human behaviors, the shared commitments and obligations arising from the need to put the collective goals before the individual self (regardless of whether the goals are set by collective decision or by the elite few). On the other hand, the commitment to and realization of a potential is instead oriented and based on the individual. We believe it is this society vs. individual orientation difference that accounts for how the competition of modal verbs reflects changes in society.

First, for the explosive increase of modal verb uses in the 1920s, we showed that the change is driven by low value modal verbs and especially the verb 可以 *kěyǐ* ‘may.’ Historically, the New Culture/May Fourth movement established the use of vernacular writing and the value of individualism and democracy. As 可以 *kěyǐ* ‘may’ is from vernacular language and rarely used in classical Chinese, the switch to writing vernacular language certainly played a role in its increased usage, among other vernacular terms. However, the question of why 可以 *kěyǐ* ‘may’ had such a drastic increase remains unanswered. Refining Leech’s original hypothesis [1] and based on our elaboration of the value of modal verbs, we argue that 可以 *kěyǐ* ‘may’ marks empowering events. 可以 *kěyǐ* ‘may’ has a range of meaning slightly wider than the English *may*. That is, in addition to granting permission, 可以 *kěyǐ* ‘may’ can also be used in affirmation of ability/potential (more like ‘are able to’). As such, the giving of permission and the affirmation of ability are two crucial aspects of empowering individuals. Generally viewed as the catalysis of modernization and democratization of China, the 1919 movement can be considered an empowering event, and a drastic increase in the use of low value modal verbs, and particularly 可以 *kěyǐ* ‘may’ could be attributed to the increasing number of empowering acts as well as reports of such events.

To explain the sustained increase of the use of modal verbs in the 1940s, we showed that it is not only driven by high value modal verbs, it also coincides with the rising share of such verbs at the cost of the low value modal verbs. We also showed that the change is largely driven by the modal verb 应 *yīng* ‘should.’ As mentioned earlier, the 1940s saw great upheaval in China including the Japanese invasion (part of War World II), and the CCP-KMT Chinese civil war. In war and in crisis, the curtailing of individualism by enforcing shared societal expectations as obligations is a necessity. Consequently, the data from that time should contain both texts enacting obligations and texts describing such events, empowering the State/collective community. Thus, the high value modal verbs are preferred. But why 应 *yīng* ‘should’ among all high value modal verbs? On the one hand, the monosyllabic words in Chinese, especially modals, are typically considered to have stronger illocutionary power due to their abruptness for being the shortest form. In addition, the monosyllabic form is considered both more classical and formal. The extreme strain on resources during World War II may have also played a role in requiring shorter texts. In sum, the change in usage tendencies of modal verbs during the 1940s is driven by the urgency at war, and most significantly manifested by the spike in the use of the monosyllabic 应 *yīng* ‘should’, which satisfied the requirements of several possible motivations for changes.

The last major changes happened in the 1950s. This change could be viewed as either the reaction to the changes in the 1940s or as the consequence of important social events such as the establishment of the PRC. The use of low modal increased significantly, at the expense of the high value modal verbs. 可以 *kěyǐ* ‘may’ contribute most to the change. The end of the war and the beginning of the PRC both could have served as the impetuses of changes.

By considering the nature and behavior of competition between low and high value modal verbs in the past century and their historical usage trends, our study was able to identify potential social-historical events that either require strong collective obligations (for high value modal verbs), or facilitate and empower individual developments (for low value modal verbs). In addition, two additional facts corroborate the account. First, interestingly, the orthography reform did not seem to have a strong effect on the use of modal verbs. A possible explanation is that the metalinguistic change does not directly affect interpersonal relations, hence there is no direct effect on the use of modality. This explanation can also be applied to other major historical events in the past century. Second, we also observed that in the decades without major change instigating events, the general usage tendency is a slow decrease with some fluctuations, of modal verbs overall, including the types that just changed. This pattern can be predicted by the hypothesis that the major changes were the consequences of major social-historical events. That is, once the memory and effect of the instigating event fade through time, the corresponding changes in modal verb usages will also fade slowly.

## Conclusion

In this paper, we report a language big data driven longitudinal study of the use of modal verbs in more than 100 years in both English and Chinese to tackle four research questions. (1) Do modal verbs in different languages share similar patterns of decline? (2) Do high, median, and low value modal verbs show different usage trends, and why? (3) Can a longitudinal study of language big data focusing on modal verbs reveal significant events and trends in history? and (4) Do modal verbs show different degrees of sensitivities to different historical events in their usage trends?

To address these theoretical issues, we adopted two innovative research designs. First, we use the largest comprehensive corpus that naturally contains a wide range of genres and varieties as determined by the accessibility of data. Our rationale is that this is the best way to resolve the size vs. balanced debate. On one hand, the Google Books corpus can be shown to be the largest dataset of the period involved. On the other hand, the criteria of collecting as much data of all kinds as possible without a pre-set quota equals a random sampling of the total data and can be treated as an approximation of the “balance” of usages that vary from time to time. Second, noting that all modal verbs are competing for the same domain of linguistic information, we added distribution of total usage in given decades as a quantitative model for usage changes and competition.

As a first step to answer these research questions and to confirm our innovative methodology over traditional “within the same corpus” studies conducted on English, we conducted our first study on English modal verbs based on the Google Books corpus but mirroring the sampling design of both Leech [1] and Millar [11]. This study is designed to reconcile the conflicting results of these two classical studies in order to underline the advantage of our new methodology driven by access to language big data. Theoretically, we also address the ongoing balance versus size debate in corpus linguistics, especially in the context of diachronic studies. By adopting the Google Books corpus, we show that the size and balance data can be optimized given sufficient sampling size. That is, given the comprehensive coverage of the Google Books corpus and its sources, its variations of composition in different time periods can be considered as roughly reflecting the usage changes of each period, and hence it can be “balanced” naturally by actual use and not artificially by a pre-determined set of criteria that are valid for a specific time but most likely not for other times.

Our internally consistent results affirm the general tendencies observed by Leech [1] and Millar [11], but with some critical differences. We showed that the overall tendencies were indeed decreasing, especially for high and median value modal verbs. Our more comprehensive data also showed that low value modal verbs do not follow this tendency and are either increasing or fluctuating. We also show that the tendencies were not uniform in the past century and did contain some periodical fluctuations. The more convincing and theoretically nuanced account this study provided showed that English modal usages reflect the social changes away from an authority-driven power structure not by just the lower usage of high and median value modal verbs, but also by the increasing usage of low value ones.

To answer the first question, we apply the same methodology to Mandarin Chinese data over the same period in our second study. Among the handful of languages with a comparable size of data continuously over the last century, Mandarin Chinese is probably the most distant from English due to both their very different language families and orthographies, but also to the more limited scale of mutual borrowing (compared with, for instance, most European languages and Japanese). In addition, China’s eventful history in the past century also provided multiple potential transition points for us to check for the correlation between modal verb usage and social changes. Interestingly, Mandarin Chinese showed several ups and downs for the overall usage of modal verbs, as well as different tendencies for different modal verbs according to their values. Crucially, we were able to establish that such changes follow impactful historical events. Thus, although the first hypothesis that modal languages in all languages show the same falling pattern is proven wrong, our data does show a correlation between the usage changes of modal verbs and social changes.

Our second research question deals with potential differences in usage trends of modal verbs according to their modality values of high, median, and low. Note that our first study based on English showed that the frequency of low value modal verbs rises while the other two types fall in Table 6. We speculated that the rise of low value modal verbs is due to the exact reason that Leech [1] suspected, i.e. the opening up of society. The Chinese data not only provide further support of correlation but also showed a more nuanced picture that challenges the somewhat simplistic view assumed by previous studies. First, our data shows that in the 1940s, when China was at war and in turmoil, an authoritative turn did reverse the direction of change, with high value modal verbs increasing and the other two values decreasing. This result reinforces our hypothesis that high and low value modal verbs could move in opposite directions. However, modal verb frequency may also slowly decrease naturally over time after a drastic rise. Hence, how to predict the overall tendencies of modal verb usage changes remain an intriguing question.

In answering the third question, the usage trends of modal verbs could both rise and fall, and the significant changes typically do reflect historical events. This study on Mandarin crucially established a tug-of-war pattern competition between high and low value modal verbs, with mirrored changes in shares of total modal verb uses through time. Theoretically, this is another non-sigmoid pattern of language changes in addition to the recently reported emergent neologism changes [53]. Strengthening Blythe & Croft’s observation [52] that the standard sigmoid function account of historical linguistics only covers a subset of replacement changes and other more comprehensive models are needed to allow us to better understand and account for the mechanisms of language changes.

The mirrored changes with reversed directions were also crucial in helping us to establish the potential correlation between usage trends of modal verbs and socio-cultural changes. Note again that without external intervention, and given the one-on-one competition, current theories would expect a replacement change following the S-curve will eventually be completed given time. This clearly did not happen. Conceptually grounded on the modality as linguistic signals on interpersonal relations, we hypothesis that these reversals arose because of the overall nature of interpersonal relation changes. The overall pattern shows that usage of low value modal verbs spike right after a critical empowering societal change (e.g. the New Culture/May Fourth movement), yet its usage tendency for a longer term is to gradually decrease. In addition, we showed that social upheaval called for curtailing of individualisms to “empower” the collective whole (at the cost of individuals) prompted the strength of usage trends of high value modal verbs. We were also able to pinpoint, in two periods of time, the single dominant modal verb that drove the changes and accounted for their prominence in terms of power relations and other criteria. Although a definite theory to predict which social changes will lead to modal verb usage changes and how is beyond the current study, we were able to combine our model of modality in terms of interpersonal relations and the nature of social changes to establish a viable hypothesis. An interesting comparison of two similar events covered by our data may provide some clues. There are two important changes in language policy and language use with contrasting influences: the New Culture Movement and orthography reform (Simplification and Latinization). As discussed earlier, the New Culture Movement empowered a large sector of the population in China by making it possible for them to write as they speak in a colloquial style [11, 54, 55]. This was a widespread enabling event, as the general public were not trained in the classical style, and therefore were seriously handicapped in their ability to read or write even for those considered to be “literate” in the sense of being able to use Chinese characters. In contrast, the orthography reform is metalinguistic by nature as it focuses on the choice of writing systems, not the basic functions of the language itself. Another point of contention is the Latinization initiative as part of the orthography reform. It is debatable whether the replacement of Chinese characters with the Latin alphabets would be empowering or disenfranchising for the uneducated population who has not learned Latin letters before. Either way, the language/orthography reform did not significantly affect the interpersonal relations of most people. Thus, there is no motivation to change the uses of modal verbs of a specific value, and no major change tendencies are observed. This result provided an answer to our fourth research question. The usage tendencies of modal verbs are most sensitive to events that have a widespread impact on interpersonal interaction.

Following Labov [20], Leech [1] initiated the data driven approach to investigate the effect of social changes on language uses, focusing on the attitudinal expression of modal verbs. Taking advantage of the availability of a larger corpus, as well as the ubiquity of language big data, this paper joins the emergent trend of using linguistic evidence to identify environmental and societal changes [8, 19, 41, 47]. In particular, this study leverages Halliday and Hasan’s theory [31] of putting language uses in the social context, and thus we can clearly identify the different behaviors of high and low value modal verbs to societal changes and we were able to produce a theoretically felicitous account that reconciles the discrepancies of the two previous studies by a careful experimental design. In this study, we also improved the methodology of language big data based diachronic analysis of a language and established a robust and accessible way to monitor changes through modal verbs as social context specific linguistic devices. We showed that this kind of longitudinal tracking of changes in language use, especially in modal verbs, is a powerful tool to monitor social and cultural changes. Our comparative analysis of English and Chinese firmly establishes the correlation between modal verb usages and societal changes. We also pointed out that recent literature erred on overgeneralization by focusing on the decreasing use of modal verbs coinciding with a society’s opening up; instead, such mutual influences are nuanced and subject to complex interaction with other factors such as cultural preferences and system-internal dynamics of the language.
